# Development and validation of a prognostic model of survival for classic heatstroke patients: a multicenter study

**DOI:** 10.1038/s41598-023-46529-7

**Published:** 2023-11-07

**Authors:** Yu Wang, Donglin Li, Zongqian Wu, Chuan Zhong, Shengjie Tang, Haiyang Hu, Pei Lin, Xianqing Yang, Jiangming Liu, Xinyi He, Haining Zhou, Fake Liu

**Affiliations:** 1 Department of Emergency Medicine, Rongxian People’s Hospital, Rongxian, 643100 China; 2 Department of Thoracic Surgery, Suining Central Hospital, Suining, 629000 China; 3 Department of Oncology, Zhongjiang County People’s Hospital, Zhongjiang, 618100 China; 4 Department of Critical Care Medicine, Jiang’an County People’s Hospital, Jiang’an, 644200 China; 5 Department of Gastrointestinal Surgery, Suining Central Hospital, Suining, 629000 China; 6 Department of Rheumatology and Immunology, Nanchong Central Hospital, Nanchong, 637000 China

**Keywords:** Epidemiology, Risk factors, Climate-change impacts

## Abstract

Classic heatstroke (CHS) is a life-threatening illness characterized by extreme hyperthermia, dysfunction of the central nervous system and multiorgan failure. Accurate predictive models are useful in the treatment decision-making process and risk stratification. This study was to develop and externally validate a prediction model of survival for hospitalized patients with CHS. In this retrospective study, we enrolled patients with CHS who were hospitalized from June 2022 to September 2022 at 3 hospitals in Southwest Sichuan (training cohort) and 1 hospital in Central Sichuan (external validation cohort). Prognostic factors were identified utilizing least absolute shrinkage and selection operator (LASSO) regression analysis and multivariate Cox regression analysis in the training cohort. A predictive model was developed based on identified prognostic factors, and a nomogram was built for visualization. The areas under the receiver operator characteristic (ROC) curves (AUCs) and the calibration curve were utilized to assess the prognostic performance of the model in both the training and external validation cohorts. The Kaplan‒Meier method was used to calculate survival rates. A total of 225 patients (median age, 74 [68–80] years) were included. Social isolation, self-care ability, comorbidities, body temperature, heart rate, Glasgow Coma Scale (GCS), procalcitonin (PCT), aspartate aminotransferase (AST) and diarrhea were found to have a significant or near-significant association with worse prognosis among hospitalized CHS patients. The AUCs of the model in the training and validation cohorts were 0.994 (95% [CI], 0.975–0.999) and 0.901 (95% [CI], 0.769–0.968), respectively. The model's prediction and actual observation demonstrated strong concordance on the calibration curve regarding 7-day survival probability. According to K‒M survival plots, there were significant differences in survival between the low-risk and high-risk groups in the training and external validation cohorts. We designed and externally validated a prognostic prediction model for CHS. This model has promising predictive performance and could be applied in clinical practice for managing patients with CHS.

## Introduction

Heatstroke (HS) is a most hazardous condition characterized by extreme hyperthermia (usually > 40.5 °C), central nervous system (CNS) dysfunction, and multiorgan failure that can be classified into two categories: classic or exertional^1,2^. Immediate alleviation of hyperthermia and support of organ system function are the main therapeutic goals in patients with HS^1,2^. The primary cause of classic heatstroke (CHS) is exposure to high temperatures and inadequate heat dissipation mechanisms^3,4^. The most prevalent populations at risk for CHS include those with compromised ability to physiologically adapt to heat stress, individuals who are unable to care for themselves, and chronically ill persons^3,4^. Heat waves, attributed to global warming and urbanization-induced inner-city heat islands, are the primary external factors that lead to an increased number of fatalities, surpassing other extreme weather events^1,4–8^. Multiple intrinsic factors, such as social, physiological, and medical burdens, make elderly individuals more vulnerable to the effects of persistent heat^3,4,9,10^. Consequently, the mortality rate from heatstroke in the elderly population is reported to be over 50%^11,12^. Additionally, critical cases of CHS increased considerably in the summer of 2022 worldwide due to unprecedented high temperatures^13,14^.

Despite the high morbidity and mortality of CHS, limited data exist regarding the clinical characteristics and prognoses of patients with this critical illness. There have been several studies on the risk factors, pathogenesis, treatment, and prevention of HS^15–18^, but little is known about how to determine the prognosis of CHS early. Anticipating patient outcomes at the point of admission can facilitate the identification of individuals at an elevated risk of unfavorable results. Consequently, these patients may receive proactive supportive treatments to enhance their prognosis. Nomograms are graphically represented mathematical models, extensively utilized to forecast prognosis^19,20^. They achieve this by estimating clinical events and incorporating key prognostic factors across a wide spectrum of diseases. Given this, a predictive model with reliable performance is crucial for the clinical management of CHS.

In this multicenter study, we aimed to identify prognostic factors from epidemiological and clinical characteristics, as well as hematological indicators, and subsequently establish and externally validate a nomogram model to predict the survival of CHS patients.

## Methods

### Study design and patients

This study was retrospective and involved enrolling patients from 4 hospitals in Sichuan Province during the period between June 2022 and September 2022. The training group comprised patients diagnosed with CHS at three hospitals in southwestern Sichuan, which were employed to establish the nomogram prognostic model. In contrast, model external validation was performed using cases from a single hospital in central Sichuan. The research adhered to the Transparent Reporting of a Multivariable Prediction Model for Individual Prognosis or Diagnosis (TRIPOD) guidelines throughout the investigation^21^.

**Figure 1:**
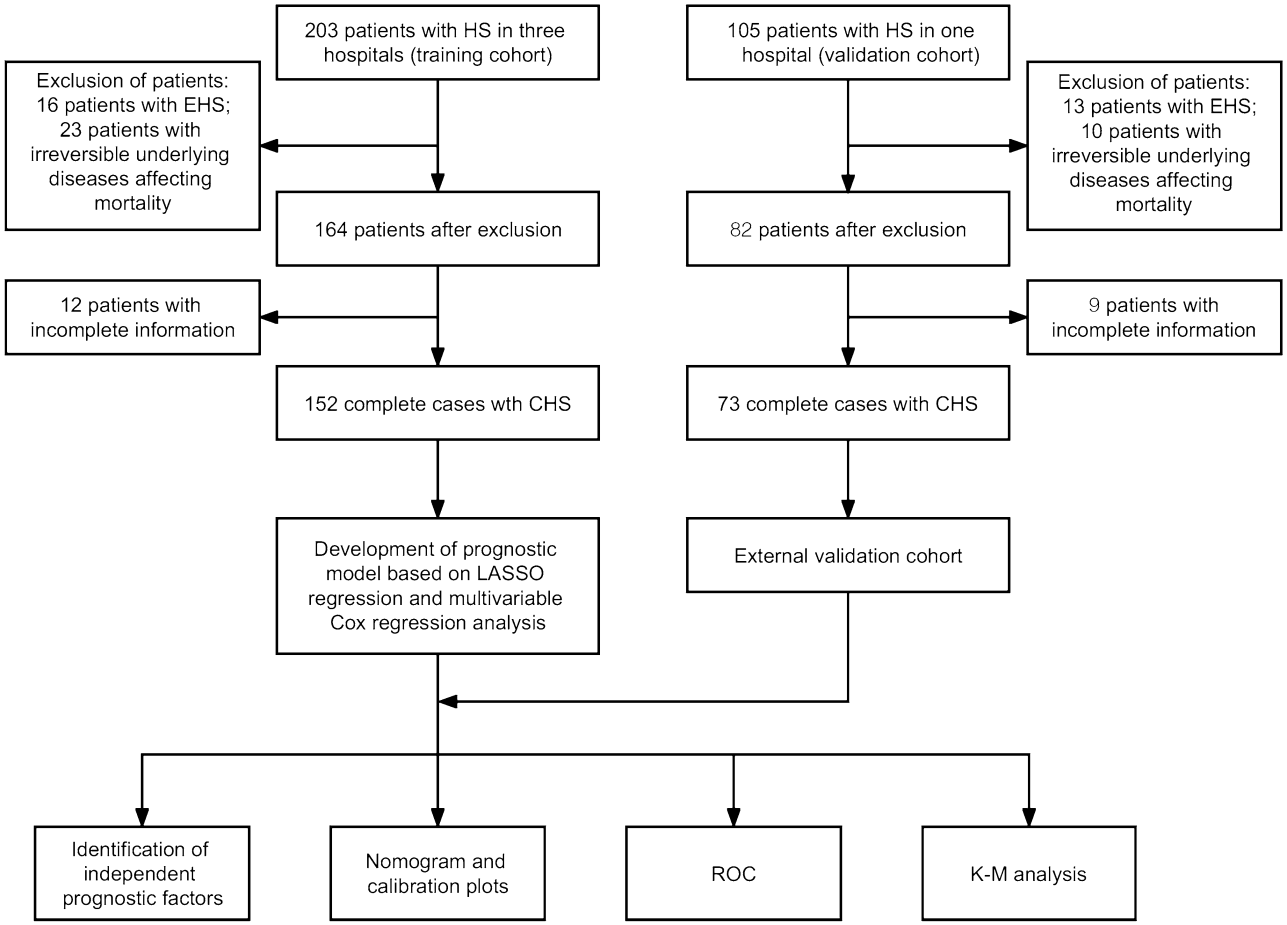
The flowchat of this study. HS: heatstroke; EHS: exertional heatstroke; CHS: classic heatstroke; LASSO: least absolute shrinkage and selection operator; ROC: receiver operator characteristic; K-M: Kaplan–Meier.

The inclusion criteria were as follows: (1) participants aged over 18 years; and (2) individuals who satisfied the CHS diagnostic criteria, which primarily focused on recent exposure to high temperature, the presence of hyperthermia and neurologic anomalies. The exclusion criteria included the following: (1) patients experiencing exertional heatstroke (EHS); (2) patients with irreversible underlying diseases affecting mortality; and (3) patients with incomplete information. The flowchart of this study is shown in Fig. 1.

### Data collection

Detailed baseline sociodemographic characteristics, clinical data, hematological indices, treatment, and outcomes were collected from the CHS patients involved. Baseline sociodemographic characteristics included factors such as sex, height, age, weight, body surface area, body mass index (BMI), smoking, alcohol, marital status, ways to hospital, unventilated and non-air-conditioned living space, self-care ability, and social isolation. Clinical data encompassed comorbidities (including hypertension, diabetes, coronary heart disease, chronic obstructive pulmonary disease [COPD] and mental illness), respiratory rate, body temperature, Glasgow Coma Scale (GCS), heart rate, blood pressure, peripheral oxygen saturation, acute respiratory distress syndrome (ARDS), brain edema, disseminated intravascular coagulation (DIC), diarrhea, multiple organ dysfunction syndrome (MODS), and hospital stay. Treatment information included antibiotic and mechanical ventilation. Outcome data, including hospital discharge or death, were also collected. Various laboratory tests, including blood analyses, were conducted to measure numerous parameters, such as blood glucose, pH, base excess (BE), HCO3-, leucocytes, neutrophils, percentage of neutrophils, red blood cells, hemoglobin, lactate, prothrombin time (PT), platelets, fibrinogen (FIB), D-dimer, activated partial thromboplastin time (APTT), procalcitonin (PCT), urea nitrogen, creatinine, troponin, myoglobin, creatine kinase MB isoenzyme (CK-MB), aspartate transaminase (AST), total bilirubin, direct bilirubin, and alanine transaminase (ALT). To reduce sampling bias, researchers communicated effectively with medical staff and double-checked the information collected with them.

According to the Ministry of Health (Ethics review on biomedical research involving human subjects), WMA (Declarations of Helsinki) and CIOMS (International ethical guidelines for biomedical research involving), all methods were performed in accordance with the relevant guidelines and regulations.

### Ethics approval and consent to participate

This study was approved by the ethics committees of the four participating hospitals, with approval numbers as follows: Rong County People's Hospital: RY2023-015, Jiang'an County People's Hospital: 2023-001-005, Zhongjiang County People's Hospital: JLSY-2023-056, and Suining Central Hospital: KYLLKS20230130. Due to the anonymous and retrospective nature of the study, the ethics committees of these four hospitals waived patient informed consent.

### Statistical analysis

Continuous variables are represented as the mean (standard deviation [SD]) when data adhered to a normal distribution, whereas the median (interquartile range [IQR]) was used in cases where the data deviated from a normal distribution. Categorical variables are presented in the form of numerical values and proportions. Survival time was determined from the patient's hospital admission until their death or discharge. LASSO regression analysis was utilized to determine potential prognostic factors in the training set. Subsequently, these identified factors underwent comprehensive analysis in a multivariate Cox proportional hazards model, aiming to discern the vital prognostic factors intimately associated with the survival of patients with CHS. Prognostically significant factors identified through multivariate Cox regression analysis were used to develop an in-hospital survival prediction model, which was visualized using a nomogram.

To examine the generalizability of the model, an external validation cohort was provided by a tertiary general hospital in central Sichuan. The model's discrimination was assessed using a receiver operating characteristic curve (ROC) and area under the ROC curve (AUC). We utilized calibration plots to evaluate the calibration of the model in both the training and validation cohorts. Apart from quantitatively evaluating the discrimination capacity through the AUC, we also endeavored to demonstrate the independent predictive ability of model at varying risk score levels. We categorized patients into different risk groups based on the overall risk score in the training cohort (from highest to lowest). The cutoff value was determined based on the median of the risk scores. Values greater than the median are labeled as high risk, while those less than the median are labeled as low risk. Subsequently, we utilized this identified value for the external validation cohort and delineated the respective Kaplan‒Meier survival curves. R software (version 3.6.3) was utilized for all statistical evaluations, and all tests adopted a significance threshold of 0.05.

## Results

### Baseline sociodemographic and clinical features of enrolled CHS patients

From the primary database of 308 patients, we excluded those diagnosed with EHS (n = 29), patients suffering from irreversible underlying diseases affecting mortality (n = 33), and cases with missing details (n = 21). Thus, in alignment with the inclusion criteria, 225 patients were ultimately included in the study. The median age of the study participants was 74 years old (IQR: 68–80 years), and 123 were male patients, accounting for 55% of the total. Most patients (81%) arrived at the hospital via ambulance, while 43 (19%) admitted themselves. The mean BMI and body surface area were 21.6 kg/m^2^ and 1.51 m^2^, respectively. Among the patients, it was common to observe living conditions without ventilation or air conditioning (71%), an incapability for self-care (27%), and social isolation (22%). The median body temperature upon admission was 40.5 ℃ (IQR: 40–41.3). Furthermore, 79% of patients had a heart rate exceeding 100 beats/min, and 17% exhibited oxygen saturation below 80% at admission. The median GCS score on admission was 8 (IQR: 5–12). The characteristics of patients in the training and validation cohorts are listed in Table 1.

**Table 1:** Demographic and clinical features, treatment and outcomes of patients in the training and validation cohorts.

Variables	Total (n = 225)	Train (n = 152)	Validation (n = 73)
Age, median (IQR), years	74 (68, 80)	77 (70, 82)	70 (64, 77)
Sex, n (%)			
Male	123 (55)	83 (55)	40 (55)
Female	102 (45)	69 (45)	33 (45)
Height, median (IQR), m	1.6 (1.53, 1.65)	1.58 (1.5, 1.64)	1.62 (1.58, 1.66)
Weight, median (IQR), Kg	53 (50, 60)	50 (45, 60)	56 (51, 62)
Body mass index (BMI), median (IQR), Kg/m^2^	21.56 (20, 23.1)	21.48(19.45, 23.55)	21.67(20.43, 22.68)
Body surface area, median (IQR), m^2^	1.51 (1.45, 1.65)	1.45 (1.35, 1.65)	1.57 (1.47, 1.69)
Smoking, n (%)			
Yes	79 (35)	64 (42)	15 (21)
No	146 (65)	88 (58)	58 (79)
Alcohol, n (%)			
Yes	54 (24)	44 (29)	10 (14)
No	171 (76)	108 (71)	63 (86)
Marital status, n (%)			
Sustained conjugal status	152 (68)	98 (64)	54 (74)
Single	73 (32)	54 (36)	19 (26)
Ways to hospital, n (%)			
Emergency	182 (81)	131 (86)	51 (70)
Other	43 (19)	21 (14)	22 (30)
Social isolation, n (%)			
Yes	50 (22)	29 (19)	21 (29)
No	175 (78)	123 (81)	52 (71)
Unventilated and non-air-conditioned living space, n (%)			
Yes	160 (71)	129 (85)	31 (42)
No	65 (29)	23 (15)	42 (58)
Self-care ability, n (%)			
Yes	163 (73)	104 (68)	59 (81)
No	62 (27)	48 (32)	14 (19)
Comorbidities, n (%)			
Yes	114 (51)	70 (46)	44 (60)
No	111 (49)	82 (54)	29 (40)
Body temperature, median (IQR), ℃	40.5 (40, 41.3)	40.25 (39.8, 41)	41 (40, 42)
Respiratory rate, n (%), breaths/min			
16–20	36 (16)	13 (9)	23 (32)
> 20	189 (84)	139 (91)	50 (68)
Heart rate, n (%), beats/min			
60–100	48 (21)	14 (9)	34 (47)
> 100	177 (79)	138 (91)	39 (53)
Initial systolic blood pressure, n (%), mmHg			
< 70	24 (11)	20 (13)	4 (5)
70–105	98 (44)	67 (44)	31 (42)
> 105	103 (46)	65 (43)	38 (52)
Peripheral oxygen saturation, n (%)			
< 80%	38 (17)	36 (24)	2 (3)
80–90%	83 (37)	66 (43)	17 (23)
> 90%	104 (46)	50 (33)	54 (74)
Glasgow Coma Scale (GCS), median (IQR)	8 (5, 12)	9 (6, 13)	6 (5, 8)
Blood glucose, median (IQR), mmol/L	10 (7.9, 12.9)	9.56 (7.97, 12.8)	10.8 (7.7, 13.95)
PH, mean ± SD	7.4 ± 0.1	7.38 ± 0.1	7.42 ± 0.1
Base excess (BE), median (IQR), mmol/L	− 3.2 (− 7.1, − 0.8)	− 4.1 (− 7.53, − 1.15)	− 1.8 (− 5.3, 1.3)
HCO3-, median (IQR), mmol/L	18.8 (15.6, 21.7)	18.8 (14.8, 21.77)	19.2 (16.3, 21.6)
Lactate, median (IQR), mmol/L	3.88 (2.1, 7.28)	5.18 (3.03, 7.37)	2.5 (1.64, 3.5)
Leucocytes, median (IQR), ×10^9^/L	11.75 (8, 15.17)	11.75 (8, 15.17)	11.7 (8.1, 15.1)
Neutrophil, median (IQR), ×10^9^/L	9.46 (6.8, 12.95)	9.46 (6.53, 12.54)	9.69 (6.86, 13.2)
Percentage of neutrophils, median (IQR)	84.6 (78, 89.3)	85.1 (79.5, 89.7)	82.3 (75, 88.2)
Red blood cells, median (IQR), ×10^12^/L	3.93 (3.56, 4.32)	3.93 (3.52, 4.26)	3.9 (3.62, 4.47)
Hemoglobin, median (IQR), g/L	121 (109, 133)	121 (108, 130.25)	125 (111, 136)
Platelets, median (IQR), ×10^9^/L	113 (73, 175)	101 (64, 162.25)	145 (102, 225)
Prothrombin time (PT), median (IQR), s	13.9 (12.9, 15.03)	13.9 (12.8, 15.05)	13.9 (13, 15.03)
Activated partial thromboplastin time (APTT), median (IQR), s	28.7 (25.45, 36.1)	26.6 (23.98, 29.63)	36.7 (32.2, 39.7)
Fibrinogen (FIB), median (IQR), g/L	2.63 (2.35, 3.16)	2.56 (2.27, 2.87)	3 (2.62, 4.01)
d.dimer, n (%), µg/ml			
≤ 0.5	51 (23)	38 (25)	13 (18)
> 0.5	174 (77)	114 (75)	60 (82)
Procalcitonin (PCT), n (%), ng/ml			
≤ 1	122 (54)	81 (53)	41 (56)
> 1	103 (46)	71 (47)	32 (44)
Urea nitrogen, median (IQR), mmol/L	7.89 (5.86, 11.67)	8.41 (6.5, 11.68)	6.78 (5.3, 9.3)
Creatinine, median (IQR), µmmol/L	112.05(70.85,161.07)	112.1(71.22,163.5)	107 (70.25, 146.25)
CK-MB, n (%), µ/L			
≤ 5	86 (38)	52 (34)	34 (47)
> 5	139 (62)	100 (66)	39 (53)
Myoglobin, n (%), ng/ml			
≤ 1000	141 (63)	96 (63)	45 (62)
> 1000	84 (37)	56 (37)	28 (38)
Troponin, n (%), ng/ml			
≤ 0.1	107 (48)	72 (47)	35 (48)
> 0.1	118 (52)	80 (53)	38 (52)
Alanine transaminase (ALT), n (%), µ/L			
7–40	121 (54)	78 (51)	43 (59)
> 40	104 (46)	74 (49)	30 (41)
Aspartate transaminase (AST), n (%), µ/L			
13–35	100 (44)	62 (41)	38 (52)
> 35	125 (56)	90 (59)	35 (48)
Total bilirubin, median (IQR), µmmol/L	19 (14.5, 25.2)	19.6 (15.8, 26.85)	16 (10, 22.8)
Direct bilirubin, median (IQR), µmmol/L	7.3 (5.3, 10.6)	7.7 (5.88, 10.33)	5.5 (3.9, 11.1)
Disseminated intravascular coagulation (DIC), n (%)			
Yes	28 (12)	25 (16)	3 (4)
No	197 (88)	127 (84)	70 (96)
Acute respiratory distress syndrome (ARDS), n (%)			
Yes	64 (28)	46 (30)	18 (25)
No	161 (72)	106 (70)	55 (75)
Multiple organ dysfunction syndrome (MODS), n (%)			
Yes	70 (31)	53 (35)	17 (23)
No	155 (69)	99 (65)	56 (77)
Brain edema, n (%)			
Yes	8 (4)	4 (3)	4 (5)
No	217 (96)	148 (97)	69 (95)
Antibiotic treatment, n (%)			
Yes	160 (71)	99 (65)	61 (84)
No	65 (29)	53 (35)	12 (16)
Mechanical ventilation, n (%)			
Yes	118 (52)	85 (56)	33 (45)
No	107 (48)	67 (44)	40 (55)
Diarrhea, n (%)			
Yes	67 (30)	42 (28)	25 (34)
No	158 (70)	110 (72)	48 (66)
Hospital stay, median (IQR), d	6 (2, 9)	4 (1, 7)	8 (5, 12)
Outcome, n (%)			
Discharged	165 (73)	112 (74)	53 (73)
Died	60 (27)	40 (26)	20 (27)

### Post-admission laboratory results

In the entire cohort, atypical laboratory results were observed, including prolonged PT and increased levels of blood glucose, creatinine, neutrophil count, and neutrophil ratio. Over half of the patients displayed elevated inflammatory biomarkers such as d-dimer, PCT, CK-MB, myoglobin, troponin, AST, and direct bilirubin (Table 1). Comparable observations were made in both the training and external validation cohorts.

### Therapeutic measures and outcomes

CHS patients received symptomatic and conservative treatment, primarily focusing on reducing hyperthermia. Among hospitalized CHS patients, the most common treatment was antibiotic therapy (71%), followed by mechanical ventilation (52%). There were 28 patients with DIC, 64 with ARDS, and 70 with MODS. Diarrhea was reported in 67 patients, and eight exhibited brain edema on imaging. The median hospital stay duration was 6 days. In the total cohort, there were 60 fatalities (40 in the training set, 20 in the validation set), and 165 patients were discharged after treatment (Table 1).

### Predictors of survival in CHS patients

**Figure 2:**
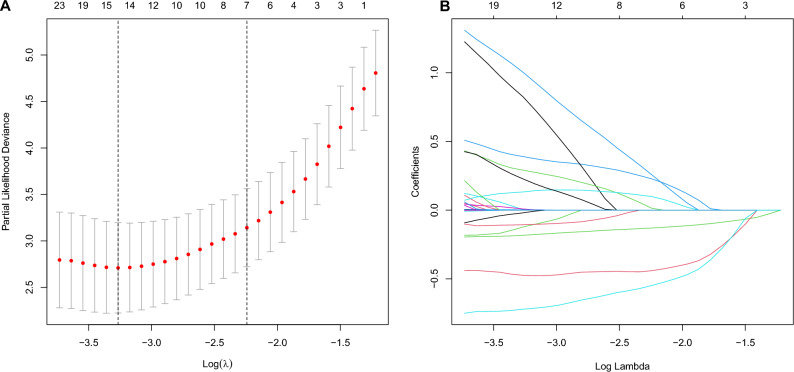
Demographic and clinical feature selection using the LASSO regression model. (**A**) Ten time cross-validation for tuning parameter selection in the LASSO model. (**B**) LASSO coefficient profiles of variables. The LASSO was employed to regress high dimensional predictors. This technique applies an L1 penalty, shrinking some regression coefficients to exactly zero. The binomial deviance curve was sketched against log (λ), where λ represents the tuning parameter. LASSO, least absolute shrinkage and selection operator.

**Figure 3:**
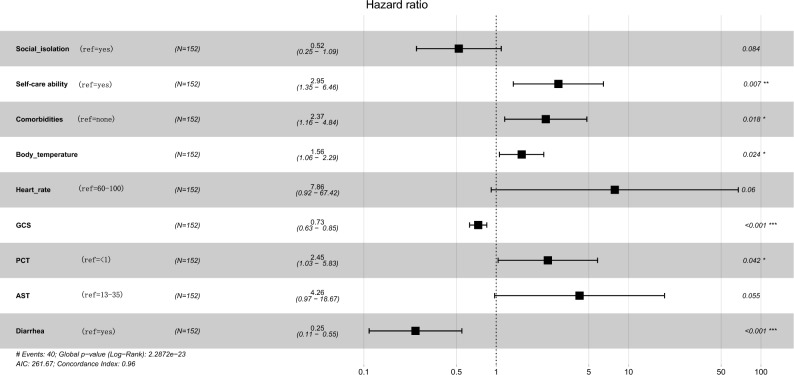
Forest map of 9 prognosis-related variables based on multivariate Cox regression. GCS: Glasgow Coma Scale; PCT: procalcitonin; AST: aspartate aminotransferase.

The baseline sociodemographic characteristics, clinical data and hematological parameter were analyzed as potential prognostic factors affecting in-hospital survival using LASSO regression. The results indicated that social isolation, self-care ability, comorbidities, body temperature, heart rate, peripheral oxygen saturation, GCS, leucocytes, FIB, PCT, CK-MB, myoglobin, AST, MODS, and diarrhea were associated with in-hospital survival at the optimal value of lambda in the training cohort (Fig. 2). To present the fitted coefficients and hazard ratios of each predictor within the model, these factors were integrated into our multivariate Cox regression analyses. Significant or near-significant associations were found between social isolation, self-care ability, comorbidities, body temperature, heart rate, GCS, PCT, AST, and diarrhea and poor outcomes in CHS hospitalized patients (Fig. 3).

### Model development

**Figure 4:**
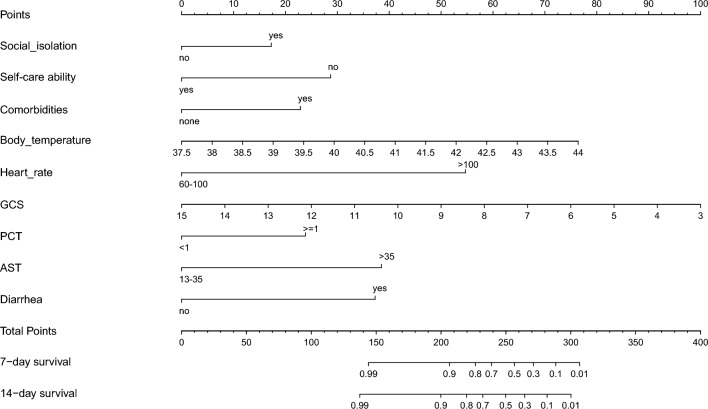
Nomogram for predicting probability of CHS in-hospital survival. The presence of each clinical characteristic indicates a certain number of points. Number of points for each clinical characteristic is on the top row. The points for each characteristic are summed together to generate a total-points score. The total points correspond to in-hospital survival probabilities. GCS: Glasgow Coma Scale; PCT: procalcitonin; AST: aspartate aminotransferase.

A predictive model was established for estimating the probability of in-hospital survival utilizing the nine independent prognostic factors identified by LASSO and multivariate Cox regression analyses. Although social isolation, heart rate and AST were borderline significant, we also included them in the construction of the nomogram, considering their clinical importance, without compromising the discriminative ability of the model. The risk score in the prognostic model for individuals was calculated by aggregating the nine variables multiplied by their corresponding coefficients: risk score = − 0.65038 × social isolation + 1.08138 × self-care ability + 0.86178 × comorbidities + 0.44265 × body temperature + 2.06185 × heart rate − 0.31373 × GCS + 0.89792 × PCT + 1.44939 × AST − 1.40371 × diarrhea. A nomogram was employed to visualize this model (Fig. 4). The nomogram demonstrated that the prognostic impact was dominated by GCS and body temperature, followed by heart rate, AST and diarrhea. Self-care ability, social isolation, comorbidities and PCT demonstrated a moderate influence on survival outcomes. On the point scale, each subcategory within these variables was assigned a score. By calculating the total score and positioning it on the total point scale, we could easily determine the estimated survival probability at each time point by drawing a vertical line.

### Calibration and validation of the nomogram

**Figure 5:**
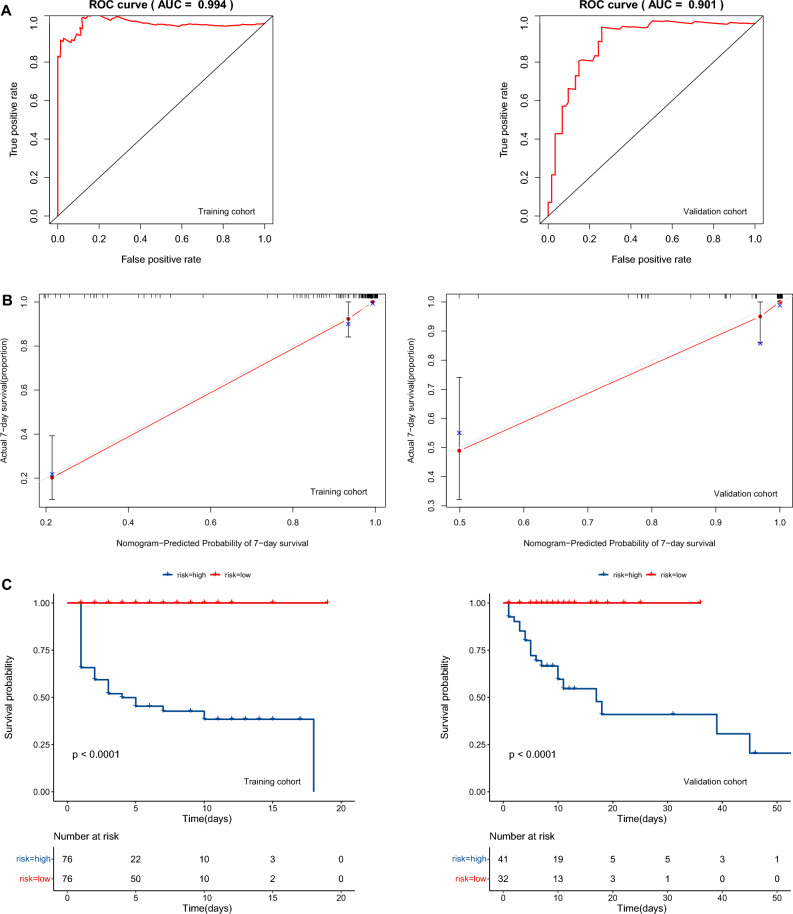
ROC curves and AUCs, Calibration plots and Kaplan–Meier survival curves of the model. (**A**) ROC curves and AUCs of the nomogram in the training and external validation cohort. (**B**) Calibration plots of the nomogram-predicted probability of 7-day survival in the training and validation cohorts. Nomogram-predicted survival is plotted on the x-axis, with observed survival on the y-axis. Dashed lines along the 45-degree line through the origin point represent the perfect calibration models in which the predicted probabilities are identical to the actual probabilities. (**C**) Kaplan–Meier survival curves of different risk groups in the training and external validation cohorts.

The predictive model's AUCs were 0.994 (95% [CI], 0.975–0.999) for the training group and 0.901 (95% [CI], 0.769–0.968) for the validation group, demonstrating the model's high discrimination capability. Calibration plots exhibited a strong concordance between the estimated 7-day survival probability and actual observations, indicating the model's good calibration (Fig. 5).

### Prognostic model risk score-based survival analyses

Based on the median cutoff of the risk score, patients were categorized into two subgroups: a low-risk group (risk score ≤ 0.778) and a high-risk group (risk score > 0.778). Survival differences between the two groups were compared using Kaplan‒Meier curves. In the training cohort, the low-risk group exhibited a significantly higher survival rate than the high-risk group. A significant (*p* < 0.001) heterogeneity was observed between the two groups in the validation cohort (Fig. 5).

## Discussion

CHS is a life-threatening condition resulting from exposure to high temperatures and inadequate heat dissipation mechanisms and has high morbidity and mortality rates, posing a substantial burden on human health and healthcare systems^1,2,22^. In this multicenter study, a prognostic model was developed and externally validated employing a substantial cohort of CHS patients. Importantly, the model allows for the incorporation of social risk factors, clinical data and hematological indices to provide personalized, patient-specific in-hospital survival estimates and can be utilized for risk stratification and prognosis analysis of CHS patients.

The primary cohort was obtained from four medical institutions from Western China. The broad geographical distribution of patients in this cohort and the consistently high temperature (exceeding 40 °C) in these regions during the previous summer ensured its representativeness and generalizability to Chinese CHS patients. Utilizing LASSO and Cox multivariable regression analysis, we identified social isolation, self-care ability, comorbidities, body temperature, heart rate, GCS, PCT, AST, and diarrhea as independent prognostic factors. Notably, although social isolation, heart rate and AST were borderline significant, we also included them in the construction of the model considering their clinical importance. Evaluated by the AUC values and calibration plots, this model exhibited strong discrimination and calibration in predicting CHS patient survival probabilities. Our multicenter study design and externally validated model shared some similarities with the previously published HS model and may offer some advantages^23^. Predictors such as HR and GCS are presented in our nomogram, which is consistent with the validated nomogram of Shao et al.^23^. However, compared to their model, ours may has several advantages. Firstly, the AUCs of both our training and validation sets are higher than theirs, indicating that our model has higher discriminative ability. Secondly, our training set encompasses a larger sample size than theirs (as the model originates from the training set), suggesting that our model might possess enhanced reliability. Furthermore, with a multitude of independent variables, there's potential for inter-variable interference. By employing Lasso regression, we are able to eliminate variables that cause such disturbances, ensuring a more robust multicollinearity among the variables.

In this study, poor prognosis of CHS was found to be associated with social isolation and inability to care for oneself. In a meta-analysis of prognostic factors for heat wave-related deaths, being bedridden, being unable to care for oneself, and not leaving home daily were associated with the highest risk of death^24^. Conversely, augmenting social connections was strongly associated with improved outcomes^24^. Elderly individuals frequently show a lower tendency to adopt protective actions, as they often underestimate their vulnerability^4,25^. Additionally, heat waves in the Northern hemisphere generally happen during holiday months when families commonly depart from urban centers, and there are many left-behind elderly people in the rural areas of western China, leaving the elderly with compromised social support. Comorbidity was another important prognostic factor found in our study. Patients with chronic obstructive pulmonary disease, cardiovascular disease, diabetes mellitus, mental disease, cerebrovascular disease, neurologic disease, and chronic kidney disease face a heightened risk of disease worsening and mortality when exposed to extreme temperatures^4^. Preexisting mental health disorders resulted in a threefold increase in the risk of death, followed by cardiovascular and respiratory illnesses^24^. Furthermore, medications such as tranquilizers, neuroleptics, diuretics, anticholinergics, and antipsychotics can reduce patients' heat tolerance; thus, these patients are at an elevated risk of inadequate water consumption, compromised thermoregulation, and mortality^26,27^.

Elevated core temperature can cause thermoregulation failure, exacerbate acute-phase responses, and alter heat shock protein expression, potentially leading to progression from heat stress to heat stroke^1^. A worsening prognosis occurs when the core body temperature consistently exceeds the critical threshold^1,28–30^. Animal model studies suggest that heat directly causes tissue damage^31^. The intensity of the injury is contingent upon the critical thermal maximum. Observations in select groups, such as marathon runners, healthy volunteers, and cancer patients undergoing whole-body hyperthermia treatment, suggest that the critical thermal maximum for humans ranges between 41.6 and 42 °C, lasting from 45 min to 8 h^32^. Routinely, people are exposed to high temperatures and cool down through an increase in heart rate, which in turn increases blood flow to the skin^22^. However, this protective mechanism increases myocardial oxygen consumption and may lead to myocardial infarction^33^. Patients with HS combined with acute myocardial infarction often have poor prognosis^33^. GCS was another significant predictor in our COX regression analysis, aligning with the findings of Shimazaki et al.^34^. The GCS score provides a straightforward and trustworthy means to assess the severity and prognosis of patients with CNS conditions. Evidence from several studies highlights that a depressed GCS suggests a poor prognosis^35–37^. Nervous system damage is particularly prominent in organ damage in heat stroke patients^1^. Brain damage appears to be focused in the cerebellum, characterized by widespread atrophy and signs of Purkinje cell layer involvement^38^. Inflammation, hypotension, and dehydration are believed to contribute to the initial stages of CNS damage resulting from heat stress^39^.

PCT is a biomarker of systemic inflammation, particularly of bacterial origin, and is useful in clinical practice for diagnosing and predicting the prognosis of bacterial infections. Although serum PCT levels typically increase in heatstroke cases, the association between PCT and heatstroke is often overlooked in clinical practice^40^. Two studies have demonstrated a connection between heatstroke and elevated serum PCT levels^41,42^. The underlying physiological link between heatstroke and procalcitonin could be mediated by the exaggerated systemic inflammatory response that characterizes heatstroke^40^. Liver injury is a common complication of CHS and a significant cause of mortality^43^. When heat stroke occurs, the blood flow of the liver decreases, and at the same time, extensive microthrombosis occurs in the liver due to concurrent DIC, causing liver ischemia and hypoxia and eventually leading to liver damage^44^. Study has reported liver injury marked by an early increase in AST and lactate dehydrogenase (LDH), peaking after 3 to 4 days, and a rise in bilirubin by the second or third day^45^. In this study, diarrhea was additionally recognized as a factor linked to unfavorable outcomes. Heatstroke can lead to gastrointestinal ischemia, which adversely affects cell viability and cell wall permeability^1^; the resulting oxidative and nitrosative stress disrupts cell membranes, allowing endotoxins and potential pathogens to enter the systemic circulation, overwhelming the detoxification capacity of the liver and resulting in endotoxemia^46,47^. Although the link between heatstroke and endotoxemia is not a new concept, many physicians tend to overlook or misunderstand applicable laboratory findings, further worsening the clinical condition and prognosis of CHS patients.

This is the first study, to our knowledge, to develop and externally validate a prognostic model for predicting the in-hospital survival of CHS patients based on social risk factors, clinical data and hematologic indices. This easy-to-use model can assist both physicians and patients in making personalized survival predictions. Validation of the model is crucial to prevent model overfitting and ensure generalizability. In this study, it's important to note that although there are differences in some variables between the training and validation sets, external validation is primarily aimed at validating and testing the model's performance, not for comparison with the training set. Therefore, it doesn't affect our results. Ultimately, the external validation cohort achieved high AUC value, and calibration plots demonstrated excellent agreement between prediction and actual observation, ensuring the repeatability and reliability of the established nomogram. Importantly, the model included patients from different levels of hospitals, supporting the nationwide application of the nomogram in both large medical centers and county hospitals. Discrimination was revealed by the significantly high AUC values of the nomogram, with only a slight reduction in the external validation cohort. Additionally, patients were stratified into high-risk and low-risk groups based on their risk scores, allowing for the identification of patients with distinct survival outcomes in the cohorts. The low-risk group exhibited significantly higher survival than the high-risk group in both training cohort and validation cohort.

Several limitations should be acknowledged. This study was retrospective with a limited sample size, which might affect the robustness of the model. Therefore, there's a need for a prospective study on a larger population to further validate our findings. The outcomes of hospitalized patients may vary depending on factors such as medical resources and economic levels. Patients with similar conditions but with a lower regional medical burden may experience better outcomes than those treated at overburdened centers. In such cases, we recommend integrating the survival predictions in the model with the actual clinical situation. Further efforts should be made to improve the model by incorporating additional factors, recruiting a more diverse and broader geographic sample, and conducting prospective data collection with long-term patient follow-up.

## Conclusion

In conclusion, we designed and externally validated a prognostic prediction model for CHS. This model has promising predictive performance and carries the potential to be applied in clinical practice for managing patients with CHS.

## Data Availability

Data sets analyzed during the current study are available from the corresponding authors upon reasonable request.

## Abbreviations

HS: Heatstroke
CHS: Classic heatstroke
EHS: Exertional heatstroke
LASSO: Least absolute shrinkage and selection operator
ROC: Receiver operator characteristic curve
AUC: The areas under the receiver operator characteristic curve
GCS: Glasgow Coma Scale
PCT: Procalcitonin
AST: Aspartate aminotransferase
K‒M: Kaplan–Meier
IQR: Interquartile range
CNS: Central nervous system
